# Vascular dysfunction in hemorrhagic viral fevers: opportunities for organotypic modeling

**DOI:** 10.1088/1758-5090/ad4c0b

**Published:** 2024-06-05

**Authors:** Evelyn Zarate-Sanchez, Steven C George, Monica L Moya, Claire Robertson

**Affiliations:** 1 Department of Biomedical Engineering, University of California, Davis, Davis, CA, United States of America; 2 Materials Engineering Division, Lawrence Livermore National Laboratory, Livermore, CA, United States of America; 3 UC Davis Comprehensive Cancer Center, Davis, CA, United States of America

**Keywords:** hemorrhagic fever viruses, microphysiological systems, vasculotropic disease

## Abstract

The hemorrhagic fever viruses (HFVs) cause severe or fatal infections in humans. Named after their common symptom hemorrhage, these viruses induce significant vascular dysfunction by affecting endothelial cells, altering immunity, and disrupting the clotting system. Despite advances in treatments, such as cytokine blocking therapies, disease modifying treatment for this class of pathogen remains elusive. Improved understanding of the pathogenesis of these infections could provide new avenues to treatment. While animal models and traditional 2D cell cultures have contributed insight into the mechanisms by which these pathogens affect the vasculature, these models fall short in replicating *in vivo* human vascular dynamics. The emergence of microphysiological systems (MPSs) offers promising avenues for modeling these complex interactions. These MPS or ‘organ-on-chip’ models present opportunities to better mimic human vascular responses and thus aid in treatment development. In this review, we explore the impact of HFV on the vasculature by causing endothelial dysfunction, blood clotting irregularities, and immune dysregulation. We highlight how existing MPS have elucidated features of HFV pathogenesis as well as discuss existing knowledge gaps and the challenges in modeling these interactions using MPS. Understanding the intricate mechanisms of vascular dysfunction caused by HFV is crucial in developing therapies not only for these infections, but also for other vasculotropic conditions like sepsis.

## 1. Introduction

The hemorrhagic fever viruses (HFVs, selected agents described in table 1) are among the deadliest pathogens facing humanity in the present era. While these viruses come from a range of families, including the arenaviruses, bunyaviruses, flaviviruses and filoviruses [1], they all adversely impact the vasculature manifesting in symptoms such as hemorrhage, edema, petechial rash, and/or bruising. With a few exceptions, these diseases lack specific treatment or preventative measures, relying primarily on supportive care for therapeutic management. Finding effective countermeasures for these infections remains an urgent issue in pandemic preparedness [2].

**Table 1.** Selected hemorrhagic fever viruses (HFV) and their interaction with the vascular system.

Virus	Viral tropism and entry mechanism	Endothelial infection & damage	Immune activation and cytokine storm	Clotting and platelet dysfunction
Filoviridae	Viral tropism	Endothelia	Immune/cytokine	Clotting
**Ebola Zaire (EBOV)** • <100 cases per year, 25%–90% mortality [3], 18% fatality rates in countries with modern health systems [4] • Presents with nonspecific fever, fatigue, and weakness with rash, followed by gastrointestinal symptoms, which can be severe and lead to hypovolemic shock [1] • ∼30% of cases develop hemorrhagic symptoms (which can be severe). • High viremia, up to 10^7 viral copies/ml blood, with no survivors showing almost 1 log greater viral copy number than survivors. [5, 6]	• Virus binds c-type lectins and other nonspecific surface glycans, internalizes through micropinocytosis and then binds NPC1 to release from endosomes [7] • Infectable cells: Endothelial cells, dendritic cells, macrophages, fibroblasts, hepatocytes, adrenal cortical cells, adipocytes, and epithelial cells [8–12].	• Direct infection and replication in endothelia observed but not associated with cytolysis [13, 14]. • Despite infection, VCAM-1 levels are not strongly increased [13, 15], which prevents leukocyte extravasation. • Tyrosine phosphorylation of CD31 on endothelia [16] • Loss of endo-BM contact [17] • Infected macrophages secrete factors that disrupt barrier function [16, 18, 19]	• Type 1 interferon response protective, EBOV able to block signaling [8] • Monocytes are infected early and traffic virus to lymph nodes [8, 20–23], • Infected monocytes/macrophages secrete high concentrations of TNF*α*, other cytokines [10, 19, 24, 25] and NO [26], • Infected PBMCs can suppress t cell activation [27] • Fatal Ebola cases lack of adaptive immunity including EBOV specific t-cells, EBOV reactive IgG, or even IgM [28–30] • Filoviruses can inhibit dendritic cell maturation [31–34] • Lymphocytes are not infectable, but undergo apoptosis, [35, 36] with declines in T cells [37] • High levels of proinflammatory cytokines Ing and TNFs IL-1*α*, IL-1RA, IL-6, MCP-1, MCSF, and MIP-1*α* associated with fatal disease [18, 19, 25, 38–41]	• Thrombocytopenia [26] • Tyrosine phosphorylation of CD31 on platelets [16] • Greater platelet function predictive of good outcome [42] • Elevated d-dimer, prolonged clotting time, high tissue factor [42, 43]. D-dimer, TM associated with fatal outcome [39] • tPA, TF, vWF, thrombomodulin increase over disease course in fatal cases [15, 38] • Elevated NO [26] • Monocytes/macrophages secrete tissue factor [44], tissue factor blockade may be therapeutic [43]
Flavaviridae	Viral tropism	Endothelia	Immune/cytokine	Clotting
**Yellow Fever (YFV)** • 5000–25 000 infections/yr [45], 30 000–60 000 deaths/yr [46]. Severe disease is is 20%–50% fatal [45] • Initial presentation of non-specific fever, malaise, and myalgia [45] • 15%–25% of cases, illness progresses to fever, vomiting, jaundice and renal failure [45] • High viremia, peaking at 10^5–10^6 viral copies/ml [45]	• Virus contains an RGD domain on protein coat- virus binds heparan sulfate and RGD binding integrins especially av3b and internalizes by membrane fusion in endosomes[45, 47–52] • Infectable cells: Hepatocytes—up to 80% of hepatocytes infected in severe disease [53], liver kupfer cells, endothelia [51, 54], spleen, heart, and kidneys, potentially central nervous system [45]. • Replicates poorly in human monocytes [55]	• Can infect endothelia [56] • Endothelial infection or NS1 from YFVF induces endothelial permeability and loss of glycocalyx [51, 54] • Entry through integrin aVb3 displaces endothelia from the BM [47] • YFV can pass through blood-brain barrier [57]	• Leukopenia and Neutropenia [45] • Elevated TNFalpha [45], IL6, RANTES [56] • Neutralizing antibodies are primary source of protection. Reinfection in humans has not been documented [45]	• Mild Thrombocytopenia [58, 59] • Loss of factors II, V, VII, IX, and X, fibrinogen [60, 61] • dysregulation of the plasmin system [60] • Elevated TT and d-Dimer seen in all cases. Fatal yellow fever associated with elevated APTT, loss of PRC, loss of FII, V, VII, IX [62]
Flavaviridae	Viral tropism	Endothelia	Immune/cytokine	Clotting
**Dengue (DENV)** • 400million cases/yr, 100million symptomatic [63], 22 000 deaths/yr [63] • Initial presentation of fever and myalgia with headache and chills, nausea and vomiting, joint pain and rash. High fever can last 2–7 d [64] • 0.5%–5% of cases progress to severe dengue, characterized by hemorrhagic manifestations including rash, bleeding, GI hemorrhage and shock • Untreated dengue fever has a mortality rate of 10%–20%, supportive care reduces to 1% • Viremia ranges from undetectable to 50% mosquito infectious dose (10^3 copies), to 10^8.5MID [64] depending on host health, serotype.	• Interacts with HS, HSP90, CD14, GRP78 and LamR, and (on myeloid cells) DC-SIGN, mannose receptor, ICAM-3, CLEC5 [65] • Can infect many cell lines *in vitro* [66], but only replicates in skin, pbmcs, spleen, lymph nodes, liver, possibly CNS, lung, and heart [67] • Monocytes, macrophages, and lymphocytes are early sites of replication [50, 51] • Possibly endothelial cells [47–52]	• Can infect endothelia, may replicate [48] causes permeability increases [49], endothelial cell ultrastructural abnormalities [68] and vWF release[69] • Cytokine tsunami (high levels of IL2, IL4, IL6, IL8, IL13 and IL18, TNF*α* and IFN*γ*) increases capillary permeability [68] • DENV reactive antibodies can cross react and damage endothelia [68] • DENV infection of endothelial cells drives destruction of basement membrane by MMP3, MMP13, MMP9 and some MMP2 [51, 54, 70, 71]	• Neutropenia and lymphocytosis [64] low leukocytes but increased lymphocyte numbers [69] • Strain mismatched antibodies can promote infection of monocytes [64] • T cells are protective but strain mismatched T cells may [72] contribute to cytokine overload • Denv infected monocytes and mast cells promote vascular leakage [68, 73] • described as a ‘cytokine tsunami’ [74, 75] with high levels of TNFa and IL6 [76, 77] causing capillary leak and pleural effusion [78–80] • MCP1 [49], VEGF and loss of plasma VEGFR2 [81, 82] • IFN signaling key to blocking DENV replication [66] • Elevated kynurenine [83]	• Thrombocytopenia [64] • DENV van activate plasminogen [84] • DENV reactive antibodies can cross react with thrombin preventing clotting and plasminogen resulting in greater fibrinolysis [85–88] • Platelet crosslinking to endothelia [58, 89] • Loss of serum serotonin [83] • DENV infection inhibits PGI-2 and ET-1, increases TM, vWF, and tPA [68] • DENV infection in children: low C, S, antithrombin III, increased TM, TF, PAI-1, TM correlated with shock severity, PAI-1 with bleeding severity [85] • Variable reduction in prothrombin, V, VII, VIII, XI, X, antithrombin and a2 antiplasmin. TF increased, no elevation in d-dimer. Slightly increased t-PA, PAI-1 and decreased TAFI, slight reduction in PC and PS [69]
Arenaviridae	Viral tropism	Endothelia	Immune/cytokine	Clotting
**Lassa (LASV)** • 100k-500k/yr [90, 91] with 5k/yr deaths [90] • Lassa fever presents with a high fever, muscle pain, nausea, sore throat, GI dysfunction, and petechial rash [92] • In 20%–30% of patients, bleeding from the mucus membranes, gastrointestinal tract, and lungs [93]. In cases with bleeding, vascular collapse, shock and acute kidney injury can lead to death [93, 94]. Deafness occurs in late stage disease or early convalescence. [92] • Viremia predicts outcome, with >10^3^ infectious particles/ml predictive of poor outcome. [92]	• Entry mechanism alpha-dystroglycan [95], or TAM, TIM or C-lecthin family receptors [96] • Monocytes/macrophages and dendritic cells are key sites of early replication and infected myeloid cells fail to activate myeloid cells [8] • Hepatocytes, endothelial cells, mucosa, connective tissues, glandular epithelia, [97]	• Increased vWF, s-p-Selectin, sICAM1, sVCAM1 [98] • Fatal Lassa associated with increased sCD31, sICAM1, sVCAM1 [99] • Binds dystroglycan strongly enough to disrupt basement membrane attachment [100]	• LFV infected dendritic cells fail to secrete proinflammatory cytokines and fail to activate T cells [8, 101, 102]. • Mixed reports of high proinflammatory cytokines [92] • Fatal Lassa fever associated with increased TNFa, sCD95, IL6, IL8, IL10, reduced sTNFaR1/R2, sCD40L, [99] • Increase in IFNg expression in infected immune cells [103] • Type 1 interferon protective, blocked by lfv infection [8]	• not associated with coagulopathy [98, 104], and clotting times remain normal in the majority of Lassa fever patients [98, 105–107]. • No thromobocytopenia to moderate thrombocytopenia reported [92, 98, 105–107] • Some evidence of higher d-Dimer [98] • Increase in sTHBS, TF, PAI, ADAMTS13 [98] • Fatal Lassa associated with increased PAI-1, sTM, tPA and antithrombin [99]
Arenaviridae	Viral tropism	Endothelia	Immune/cytokine	Clotting
**Junin/Argentine hemorrhagic fever (JUNV)** • 300–1 k cases/yr [108] • 10%–30% case fatality rate • Presents with malaise, anorexia, chills, headache, myalgia, and fever, progressing in 20%–30% of cases to neurologic-hemorrhagic manifestations [109] • Viremia present throughout infection [109]	• Entry mechanism transferrin receptor 1 through clathrin mediated endocytosis [110] • Can infect endothelium, myocardium, kidneys, CNS, [109] • Infect and replicate in Monocytes, Megakaryocytes [111] • Can infect and replicate in endos with minimal cytopathy [110, 112–114]	• Can infect and replicate in endos, with minimal [112, 113] to mild [110, 114] observed cytopathic effects • Enhanced expression of VCAM1, ICAM1, DAF, NOS, PGI3 release, loss of vWF [110] • re	• Leukopenia [109, 115] • Elevated IL6,8,10, TNFalpha [109], • Extremely high IFNalpha [116, 117] • Reduced complement hemolytic activity [110, 118] • High IFNa titer,	• Thrombocytopenia [109, 115, 119] due to megakaryocyte dysfunction [110] • Elevated clotting time [110], • Elevated d-dimer [110], but normal FDP • elevated NO, PGI2 [113], increased plasminogen [120], • prolongation of activated partial thromboplastin time (APTT), low levels of factors VIII, and IX; increased values of factor V, von Willebrand factor, and fibrinogen; decreases in antithrombin III and plasminogen [109]
Bunyavirales	Viral tropism	Endothelia	Immune/cytokine	Clotting
**Sin Nombre Hantavirus (SNV)** • 30 cases/yr [121] • Mortality of >75% [122], >30% with treatment [123, 124] • Presents with fever, myalgia and GI symptoms. • Lung edema due to endothelial infection, damage [125, 126] • Pulmonary edema develops and leads to cardiogenic shock [127] • High viremia during infection- 10^6 ml^−1^ [128]	• SNHV entry requires PCDH1 [129] • SNHV can bind inactive and active avB3 integrin [130] entry mechanism through an endosomal uptake pathway • Can infect and primarily replicate in endothelia, especially those of the lung [123, 131] • Monocyte/macrophage/dendritic cells [132]	• Endothelial cells infectable without cytopathic effects., [133] • Entry thru aVb3 perturbs barrier function by blocking BM binding and increasing VEGF-VEGRF2 signaling [134, 135] resulting in loss of VE-Cadherin [122] • Hyper VEGF causes vascular permeability [136–138] • SNV infected endothelia secrete RANTES, IP10 [133] • Binds integrin aVb3—unclear if this displaces basement membrane adhesion but affects binding to vitronectin [130, 135, 139, 140]	• Suppression of IFN [122] • Higher numbers of SNV reactive CD8 T cells in severe cases [122] • SNV reactive CD8T cells promote endothelial permeability [122, 131] • Monocytes/macrophages and dendritic cells infectable [133] • Increased IFNa, IL1a, IL-6 & TNFa [141, 142]	• Thrombocytopenia[122], associated with poor outcome [143] • Elevated clotting time [125] • Virus mediates crosslinking of platelets to endos [130] • 30–100x increase in PAI-1 in fatal cases [144, 145]
Bunyavirales	Viral tropism	Endothelia	Immune/cytokine	Clotting
**Puumala virus (PUUV)** • 150kcases/yr [146] • Mortality of 0.08%–0.4% [123, 146] • Up to 90% of infections are asymptomatic, infection shows extensive variation [147, 148] • Presents with high fever, malaise, headache, abdominal and back pain, dizziness, vomiting, blurred vision, thrombocytopenia, proteinuria, haematuria and transient renal failure [149] • Lower viremia 10^4^–10^6^ [150]	• Entry through avB3 integrin [151] through clathrin dependent endocytosis [123]. Viral binding to cd55 permits infection from apical surface possibly by altering junctional complexes and increasing permeability [151] • Can infect and primarily replicate in endothelia, especially those in the kidney [123, 132] • Monocytes/macrophages [132] • CD8+ T cells may be susceptible to infection [152]	• Increases kidney endothelial permeability and vasodilation [153, 154] • Bradykinin system disregulation [155] • Increased TNF*α*, NO, TNFalpha, IL1, IL6, IL10 [123, 156–158] promote vascular permeability • Binds integrin aVb3—unclear if this displaces basement membrane adhesion [135, 139, 140]	• Increased TNF*α*, NO, TNFalpha, IL1, IL6, IL10 [123, 156–158] • Recruitment of CD8+ CTLs to kidney [123] • Expansion of NK cells [159] • Increased serum IDO [123]	• Thrombocytopenia [160, 161] • Elevated vWF, fibrinogen and fibrin [123] • DIC, elevated d-dimer [160, 161] decreased fibrinogen [160] • Activated complement system correlates with disease severity [123]
Bunyavirales	Viral tropism	Endothelia	Immune/cytokine	Clotting
**Crimean Congo Hemorrhagic Fever virus (CCHFV)** • 11k cases reported/yr [162] • Case fatality rate of 4.7% [163] with rates as high as 70% reported [164] • High viremia in early phase of disease e6–e9 viral copies/ml [165] & in fatal cases e8–e10 [166, 167] • Presents with high fever, chills, headache, dizziness, back and abdominal pain [164] • Cerebral hemorrhage and neuropsychiatric changes have been reported [164] • In severe disease, hemorrhagic manifestations including petechiae, bleeding [164]	• Entry by binding to cell surface nucleolin [168] and/or DC-SIGN [169]and internalization through clathrin mediated endocytosis • Can infect Monocytes/macrophages	• Capillary fragility [164] • Elevated endocan indicates endotheliitis [170]	• PB monocytes show dysregulated interferon signaling [163]	• Severe thrombocytopenia [171–174] • DIC, with grossly elevated aPTT, PT, FDPs [164, 170, 172, 174] • Antithrombin iii, PS, PC below normal range in subset of patients, d-dimer elevated in majority of patients. • Low platelet count, longer PT and aPTT, elevated fibrinogen associated with mortality [174]
Bunyavirales	Viral tropism	Endothelia	Immune/cytokine	Clotting
**Rift valley fever (RVFV)** • Occasional epidemics infecting between a few hundred and 200k people [175] • Case fatality rate of ∼1% but 50% mortality rate in severe systemic disease patients [175] • Presents with sudden onset headache and body aches, followed by fever. GI symptoms, headache, backache, vertigo, anorexia can also develop [176] Eye problems in 10% of patients [175] • Severe rift valley fever can encompass liver damage and hemorrhagic disease, ocular disease or encephalitis [177]	• Internalization involves heparan sulfate on cell surface [178], possibly DC-SIGN [179] • Infects vascular smooth muscle cells, endothelial cells and tissue macrophages, replication in human monocytes [179]	• Fatal cases associated with elevated E-sel, TM, ICAM [180]	• Leukopenia [176] • 1/3 of affected patients IgM negative [176] • Loss of splenic t lymphocytes [179]	• Thrombocytopenia in a subset of patients [176] • Associated with fatal outcome: elevated d-dimer, elevated TM [180] • Correlated with viral load: tPA, d-dimer, anticorrelated: fibrinogen [181]
Henipaviruses	Viral tropism	Endothelia	Immune/cytokine	Clotting
**Nipah Virus (NiV)** • heart, kindey and CNS [182] • <1 k cases reported [183] • Mortality 30%–90% [183, 184] • Presents with fever, headache, dizziness and vomiting, Develops into severe encephalitis [183] and vascular inflammation and thrombosis in lung,	• Entry through EphB2 [185, 186], potentially also through nectin-4 [182], blocked by gal1 [187] • Can infect and replicate in endothelia, with preference for arterial endothelial cells [188] • Macrophages, [182] • Airway epithelia [189] • Can infect and replicate in smooth muscle without cytopathy [190]	• Infection of endothelia does not result in IFN response or immune adhesion receptors [188] • Infection induces arterial endothelial fusion into syncytia, blocks replication, and results in increased permeability and cell death [188, 191, 192]	• Disproportionate production of TNFa and IL1B • Recovery is associated with viral specific IgG [193]	
Coronaviruses	Viral tropism	Endothelia	Immune/cytokine	Clotting
Severe acute respiratory syndrome coronavirus 2 (Sars-CoVCV-2) • In the US, over 100 K confirmed cases since the pandemic started [194] • Viremia associated to SARS-CoV-2 mortality [195]	• Pulmonary and bronchial epithelial cells • Cells in gastrointestinal tract, kidney, heart muscle, and other organs [196]	• Induces fusion of endothelia into syncytia [197] • Spike protein and nucleocapsid activate endothelial cells [198]	• Cytokine storm • Lymphopenia in severe cases [199] • Tcell exhaustion and dendritic cell deficiencies [200–202] • Immune activation from neutrophils and complement [203, 204]	• SARS-CoV-2 genome found in platelets from patient suggest direct infections [205] • High D-dimer levels associated with development of venous thromboembolism [206, 207] and poor clinical outcome [208, 209]

*Note:* Bold indicates the pathogen name

While the specific mechanistic actions of this group of pathogens on vascular function varies widely, common features of the pathology include the triad of (1) disruption of the vascular endothelial barrier; (2) derangement of blood clotting; and (3) immune dysregulation. This vascular triad is intricately linked: altering one element impacts the others [210]. Thus, understanding the mechanisms of vascular dysfunction in HFV may provide therapeutic strategies applicable to a range of vascular diseases.

There is an acute need for an improved understanding of underlying vascular dysfunction mechanisms associated with HFV. Moreover, there is a demand for the development of disease-modifying therapies to manage vascular dysfunction in HFV and other vasculitis-associated clinical conditions such as sepsis. Anticoagulants, fluid resuscitation, and supportive care are mainstays of therapy. Despite the approval of cytokine modifying therapies for other conditions (such as cytokine blocking antibodies for IL6, TNF*α*, or VEGF), clinical trials of these therapies have not been successful in treating infectious infection-related vascular conditions (such as a septicemia), highlighting the challenges in treating vascular dysfunction. More fundamentally, this lack of clinical success indicates an incomplete understanding of these diseases due, in part, to insufficient model systems used to study these conditions (e.g. animal models or 2D cell cultures) that only partially recapitulate the underlying pathology of the vasculature.

Modeling the pathogenesis of these infections requires replicating the complex interaction of the endothelium, the clotting cascade, and the immune system. While animal models have contributed invaluable understanding of these pathogens, they are stymied by well-documented species differences in viral susceptibility and pathogenesis. Although human cell cultures mimic some elements of human vascular biology, they lack the complete functionality of *in vivo* endothelia, which depends on microenvironmental factors such as vascular flow. The emergence and adoption of microphysiological systems (MPSs) [211]) (e.g. flow cells, ‘organ-on-chip’ vasculature, bioprinted vascular beds), prompts us to explore how we can model the vascular triad. This includes investigating if existing models could be used for treatment development and identifying the specific elements necessary for integration into next-generation MPS systems to accurately model this vascular triad.

**Figure 1.**
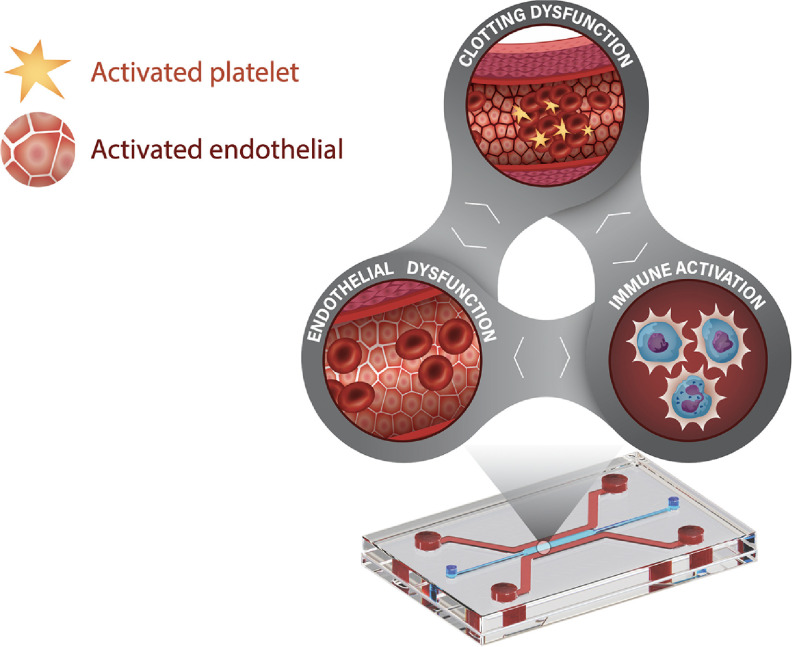
Modeling the vascular triad of hemorrhagic fever viruses on a chip. Hemorrhagic fever viruses (HFVs) derive from a range of families including the arenaviruses, bunyaviruses, flaviviruses and filoviruses. They share common symptoms associated with vascular dysfunction which can be encompassed in the ‘vascular triad’: (1) clotting dysfunction; (2) immune dysregulation; and (3) endothelial dysfunction. Each component of the triad can individually contribute to hemorrhage, but can also interact and augment other components of the triad thus complicating treatment strategies.

Our goal in this review is to discuss how HFV infections induce severe vasculitis through their effects on endothelial, clotting and immunity, identify gaps in knowledge, and outline current progress and challenges in modeling these infections *in vitro*. Specifically, we will explore the complex interplay between endothelial cells, blood clotting, and immune dysregulation and highlight unique challenges in modeling these interactions using MPS (figure 1). Lastly, we will discuss the state of vascular MPS and how these next-generation *in vitro* models can provide mechanistic insight on the pathogenesis of HFV.

## 2. Endothelium

**Figure 2.**
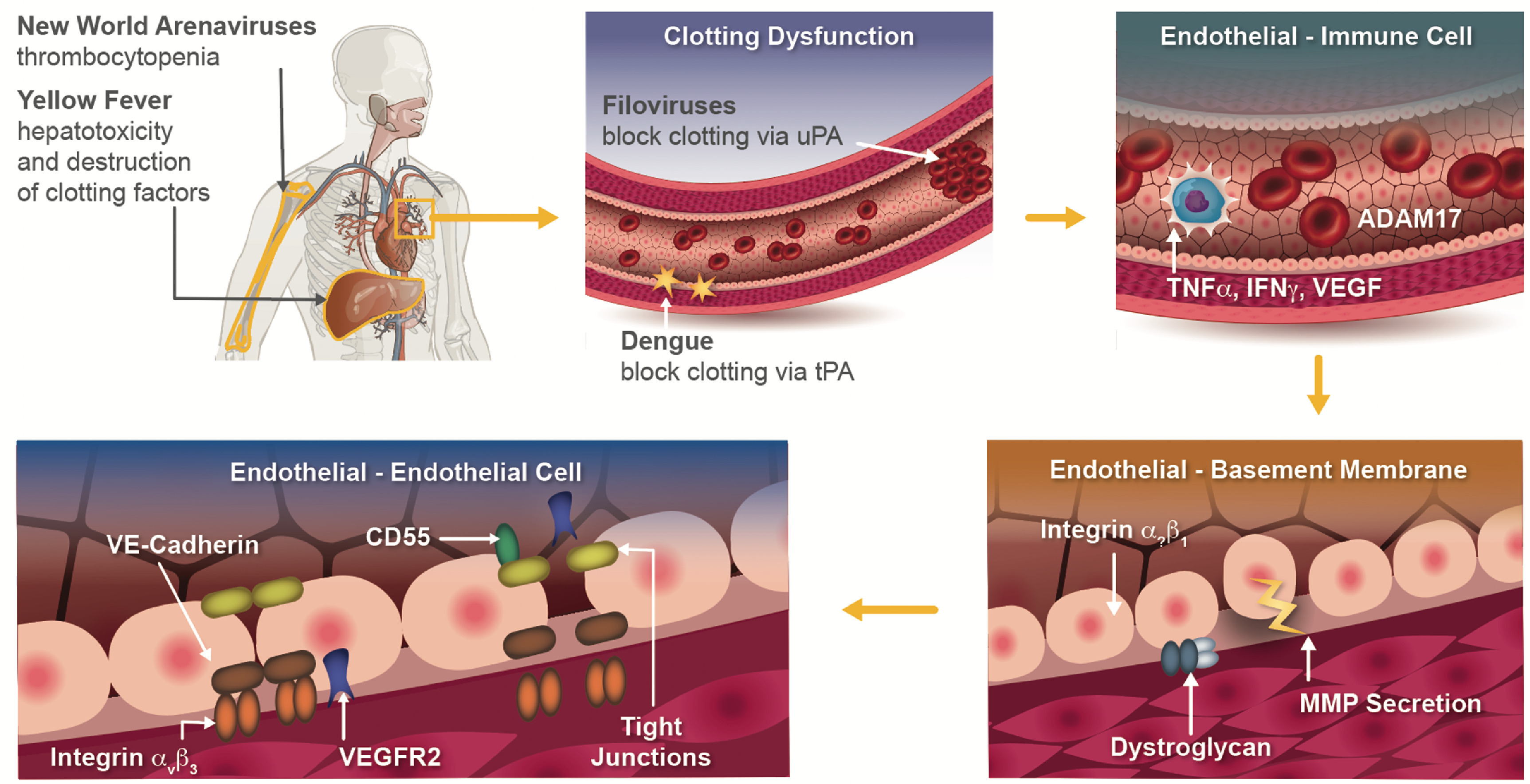
Mechanisms of hemorrhagic fever viruses. Hemorrhagic fever viruses (HFVs) encompass a family of viruses characterized by vascular dysfunction and bleeding (i.e. hemorrhage). Some HFV induce hemorrhage indirectly by impacting organs such as the bone marrow and liver that are the source of cells which produce platelets (megakaryocytes) and clotting factors, respectively. Other viruses directly impact the cardiovascular system, and more specifically the endothelium, through a host of mechanisms that include inhibition of clotting, stimulation of immune cells and secretion of inflammatory mediators (e.g. TNF*α*), degradation of the basement membrane through endothelial cell secretion of enzymes, and disruption of endothelial cell–cell junctional proteins. uPA: urokinase plasminogen activator; tPA: tissue plasminogen activator; TNF*α*: tumor necrosis factor-alpha; IFN*γ*: interferon gamma; VEGF: vascular endothelial growth factor; MMP: matrix metalloprotease; VEGFR2: vascular endothelial growth factor receptor two.

Hemorrhagic fevers are known for disrupting vascular integrity, through a wide array of mechanisms (figure 2). Thus, it is not surprising that the pathogens considered in this review almost universally share the ability to infect endothelial cells, although their impact on the endothelium varies significantly. Cultured endothelial cells infected with Sin Nombre hantavirus (SNV) or Ebola virus (EBOV) show minimal to no cytopathic effects [13], whereas Nipah virus (NIH) and Dengue virus (DENV) induce cell death by syncytial fusion, and reactive oxygen species (ROS) production respectively [28]. While infection with any of these viruses results in dysfunctional endothelium, these viruses show markedly different mechanisms for inducing endothelial dysfunction, which can include disruption of the glycocalyx, intercellular junctional complexes, and basement membrane (BM) (figure 2).

### 2.1. Glycocalyx

High viral titer in the blood (viremia) and systemic dissemination through the vasculature are common features of these viruses. In healthy endothelial cells, the apical glycocalyx provides the first line of defense against pathogens circulating through the vasculature. The glycocalyx is comprised of negatively charged glycoproteins, including hyaluronan, chondroitin sulfates and syndecans, and typically repels cells and pathogens, sequesters clotting enzymes, and masks leukocyte adhesion molecules. Disruption of the glycocalyx comprises endothelial sensing and can promote endothelial hyperpermeability independently of cytokine signaling [29, 30], a breakdown phenomenon observed in a range of infections [31]. Moreover, enzymes responsible for glycocalyx degradation are upregulated in patients infected with DENV [212], hemorrhagic fever with renal syndrome [213] and most recently, in Sars-CoV-2 infection [214]. In infections like yellow fever virus (YFV), Rift Valley fever (RVFV) and DENV [47–52, 178, 215], partially degraded glycocalyx, particularly heparan sulfates, might contribute to viral attachment to the endothelium and subsequent infection. This degradation permits pathogens to direct access to entry receptors on the apical surface of endothelial cells [216].

### 2.2. Endothelial junctional complexes

In healthy vessels, endothelial cells are tightly connected through cell–cell junctions including tight junctions, gap junctions, and adherens junctions [217]. HFVs have developed a wide toolbox of mechanisms to disrupt these junctional complexes. Importantly, endothelial cells are polarized, where the apical surface (facing the lumen) and the basal surface (facing the BM) display different receptors. There is growing appreciation that some HFVs, including Crimean Congo Hemorrhagic Fever (CCHFV) [218, 219], Puumala orthohantavirus (PUUV), Lassa virus (LASV) [70], possibly EBOV [220, 221] show preferential entry into cells from the basolateral surface. Infection thus requires the virus to extravasate through a tightly controlled monolayer designed to exclude viruses. (Note this is not universal; Nipah virus (NiV) [222], Andes hantavirus (ANDV) [223] can infect from either side of the endothelium).

As passing through the endothelium is often necessary for infection, these viruses have developed multiple mechanisms to promote endothelial permeability. DAF (CD55) represents a common binding point for various bloodborne viruses, including PUUV [151]. When PUUV or other hantaviruses bind to DAF and infect cells, it induces remodeling of tight junctions [224]. Subsequently, the virus is transported to the basal surface where it internalizes through *α*_v_ *β*_3_ integrin [151]. In the case of DENV, the virus uses a target cell’s enzymatic capabilities to degrade tight junctions: the NS1 protein from DENV tethers MMP9 to junctional complexes resulting in enzymatic degradation of ZO1/2 and beta catenin [225]. Additionally, cytokines found in serum of patients infected with DENV or other HFV, impact the expression and turnover of tight junction proteins [226], resulting in increased vascular permeability.

CD31 (PECAM-1) plays a role in cell–cell junctional complexes through modulation of STAT tyrosine phosphorylation and nuclear localization. EBOV and other filoviruses can induce tyrosine phosphorylation of CD31 both directly and indirectly by triggering infected monocyte secretion of cytokines [16], reducing endothelial cell–cell adhesion and promoting heterotypic leukocyte-endothelial or platelet-endothelial adhesion.

### 2.3. Basement membrane (BM)

Endothelial cell polarity requires attachment to the subtending proteins of the basement membrane (BM). The BM is susceptible to enzymatic degradation: elevated MMPs are observed in patient serum in CCHFV [227] and DENV [51, 54, 228], in dendritic cell supernatants infected with ANDV [71], and in serum of animals infected with Rift valley fever virus (RVFV) [229]. This enzymatic attack can degrade BM components (and possibly the glycocalyx and junctional complexes) and increase vascular permeability.

Disrupting endothelial-BM adherence can also occur through viral interactions with cellular receptors for BM proteins. For example Lassa fever virus (LFV) and other old-world arenaviruses have such a strong affinity for *α*-dystroglycan that viral particles can detach cells connected to the BM, resulting in increased endothelial permeability [230]. The NY-1, SNV, Andes and Maporal hantaviruses use integrin *β*_3_ as their entry mechanism [134, 231–233], binding it with high affinity [134]. However, the mechanism of hantavirus-integrin binding is distinct from the native mechanism of integrin-RGD ligand binding [134, 234]. Interestingly, nonpathogenic hantaviruses bind a different RGD binding integrin, *α*_5_ *β*_1_ [141] and do not induce endothelial permeability. The binding of pathogenic hantaviruses to *α*_v_ *β*_3_ integrin regulates cellular localization of receptors including PDGRF and VEGFR2, and their sensitivity for their ligands [114, 235]. Specifically, the cell–cell junction protein VE-Cadherin is anchored to the basolateral cell surface of endothelial cells by *α*_v_ *β*_3_ and in turn helps sequester VEGF-R2 as an element of contact inhibition. When *β*_3_ integrin is inhibited, VE-Cadherin junctions disassemble, reducing localization of VE-cadherin and catenins to the cell–cell junctions [114], and VEGF-R2 phosphorylates, resulting in increased sensitivity to VEGF, one of the most potent vascular permeability factors [136–138].

### 2.4. Endothelial cell activation

Once viruses have entered cells, the next barriers to infection and replication are the multiple mechanisms contained in the endothelium to sense infection and recruit and activate leukocytes (which we discuss in later sections) [236, 237]. In HFVs, endothelial activation and surface expression of immune recruitment proteins (VCAM1, ICAMs, selectins) can lead to different outcomes: suppression, resulting in lack of immune response [238], presence leading to activation and extravasation of lymphocytes [24, 239], or abnormally high expression resulting in heightened T cell adhesion and activation [133]. Indeed, the response of cells to infection can be counterproductive: nitric oxide (NO) release represents another anti-infective mechanism which is dysregulated in HFV infection [13]. NO can destroy pathogens, but in excess it promotes vasodilation, capillary leak, and shock.

### 2.5. Endothelial cell heterogeneity

Lastly, endothelial cells are not a homogenous population [240], with arterial, venous, and capillary endothelia from different organs exhibiting different susceptibility to virus infections. For example, arterial cells are susceptible to infection by Nipah and Hendra viruses as arterial cells express higher levels of the entry receptor used by these viruses, EFNB2 [188]. The flavivirus NS1 protein interacts with glycocalyx proteins including CS and HS, leading to a preference for microvasculature over larger vessels [241]. Importantly, different flaviviruses exhibit varying tissue tropism in humans: NS1 proteins from different filoviruses induce increased endothelial permeability *in vitro* in cells known to be infected in patients (i.e., Zika shows tropism for brain microvasculature, YFV for liver) [242]. Looking forward, advancements in techniques now offer a deeper understanding of cellular diversity within the vascular compartment; we expect in the future to see improved understanding of how vascular diversity interacts with pathogen response.

## 3. Coagulopathy

The pathogenesis of HFVs is intimately associated with coagulation; these pathogens are defined by hemorrhagic manifestations of bleeding, bruising, and plasma volume loss. Loss of platelets is found in EBOV, YFV, DFV, Junin (JV), SNV, PUUV, CCHFV [26, 58, 64, 109, 115, 119, 122, 160, 161, 171, 172], and frank disseminated intravascular coagulation [243] is seen in CCHFV and PUUV [160, 161, 170, 172]. The exception among HFVs reviewed here is LFV [98, 104–107]. LFV causes bleeding through direct damage to vascular integrity, which eventually results in coagulation dysfunction.

While a full discussion of coagulation is beyond the scope of this review (see [244]), key upstream triggers of coagulation are tissue factor release by damaged cells, platelet activation, high molecular weight kininogen release, or contact of factor XII to an abnormal surface. Each of these unique triggers activates a shared cascade that activates thrombin to cleave soluble profibrinogen, resulting in formation of insoluble fibrin. This process is opposed by anticoagulants such as thrombomodulin, activated protein C and antithrombin III. Once fibrin is cleaved, it can be crosslinked into a more stable clot, which can then be broken down by the plasmin system (activated by tissue plasminogen activator, tPA, or urokinase plasminogen activator, uPA) into degradation products including fibrin d-dimer.

### 3.1. The clotting cascade

Healthy endothelia oppose coagulation: thrombomodulin on the surface of the endothelia can bind and sequester active thrombin; this complex takes on anticoagulant properties including activating protein C, which in concert with its cofactor protein S, blocks several elements of the coagulation cascade, and tissue factor pathway inhibitor, which opposes the initiation and propagation of the coagulation cascade. Endothelial cells also produce nitric oxide and prostaglandins which block platelet activation. Damage to endothelia releases von Willebrand factor (vWF), which cleaves thrombomodulin from the surface of the endothelium, stabilizes factor VIII, contributes to platelet activation and mediates platelet crosslinking to the damaged matrix (which is opposed by the enzyme ADAMTS13 derived from the liver). Elevated vWF is found in EBOV, especially fatal cases [15, 38] and PUUV [123]. In addition, thrombomodulin release into the bloodstream is observed in fatal EBOV [39], DENV [68, 85], fatal LFV [99], and RVFV [180], consistent with both damage to endothelia and a procoagulant state.

Fibrin degradation products such as fibrin d-dimer are found in all the viral infections discussed in this work [69, 98, 110], indicating that patients infected with these viruses are both actively forming clots and breaking them down. Plasminogen is typically activated by tPA or uPA into plasmin, which breaks down fibrin. Infection with EBOV, LFV or DENV is associated with elevated tPA and uPA [15, 38, 69, 99]. Plasminogen can also be directly activated by DENV virus in the serum [84], or by cross reactive DENV antibodies [85–88, 245], resulting in aberrant fibrinolysis. Plasminogen activation inhibitor concentrations, which decrease fibrinolysis, can also be massively increased: PAI-1 can be increased 30–100 fold in fatal SNV infection [144, 145], and 12–20 fold in fatal LFV [99].

### 3.2. Clotting factor synthesis in the liver

Derangement of the clotting system in HFV infections can start in the liver. Hepatocytes secrete fibrinogen, prothrombin, factors V, VII, IX, X, XI, XII, protein C, protein S, antithrombin iii, and ADAMTS13, and liver sinusoidal endothelia produce factor VIII and vWF [246]. Although various HFVs discussed in this work exhibit some level of liver damage, the pathogenesis of YFV originates in the liver (with as many as 80% of hepatocytes infected [53]). YFV infection blocks hepatic synthesis of clotting factors II, V, VII, IX and X and fibrinogen [60], dysregulates the plasmin system [61] and promotes consumption of coagulation factors [247]. The derangement of clotting factor synthesis appears unique to YFV. Interestingly, the liver endothelium may play an outsized role in hepatic dysfunction in yellow fever: direct infection of hepatocytes by YFV does not induce coagulation abnormalities [247], whereas the inflammatory cytokine response triggered by YFV infection of the hepatic endothelium does block synthesis of the necessary coagulation factors [248].

### 3.3. Platelets

Loss of platelets is found in EBOV, YFV, DFV, Junin (JV), SNV, PUUV, and CCHFV [26, 58, 64, 109, 115, 119, 122, 160, 161, 171, 172]. Low platelet numbers can arise from either reduced production or increased consumption: both mechanisms are seen in HFVs. Megakaryocytes, which produce platelets, are susceptible to infection by JV [110, 111] and DENV [69, 249] resulting in loss of platelet production, whereas PUUV can infect megakaryocytes but without effects on platelet genesis [250]. Platelet consumption by crosslinking to endothelia appears a common factor in DENV [58, 69, 89] and SNV [130], but other mechanisms of consumption exist. DENV infected monocytes could contribute to platelet loss by phagocytosis [251]. For example, autoreactive DENV antibodies have been shown to bind platelets, resulting in platelet loss [252]. Likewise, Hantaviral binding to *α*_v_ *β*_3_ and *α*_IIb_ *β*_3_ receptors can induce platelet hantaviral infection and platelet damage. Serotonin loss was noted to be predictive of DENV and the most dramatically reduced metabolite in the serum of these patients [83]. Serotonin is essential for platelet aggregation and its loss suggests a dysfunctional platelet state. Loss of platelets results in delays to appropriate clotting and lack of clot integrity.

## 4. Immune dysregulation

The immune dysregulation seen in HFV infections can involve both early immune hypoactivity permitting viral spread and late hyperactivity resulting in bystander damage to tissues. Lymphocytopenia is found uniformly in these infections though these viruses almost uniformly do not infect lymphocytes (SNV is the sole exception considered in this work [152]). Suppression of immunity arises from both the suppression of the interferon system and effects on myeloid cells which permit, and even promote viral spread and replication early in infection.

### 4.1. Interferon system suppression

Dysregulated immunity starts in non-immune cells. Free cytoplasmic RNA from infection by HFVs should activate the interferon system [253], yet HFVs are known to suppress expression of interferons (IFNs). VP35 in EBOV [254], the Gn protein in ANDV, NSs protein in RFVF [255] the NSs protein in RFVF [255], NP in LFV [256] and sfRNA in strains of DENV [257] all show the ability to suppress or shift interferon expression. Indeed, failure to develop an early interferon response distinguishes HFVs from their non-pathogenic relatives, and interferon knockout mice have demonstrated utility as susceptible animal models [8, 66, 258–261].

The exceptions to this pattern of interferon suppression are the new world arenaviruses. Patients with Argentine hemorrhagic fever infections have shown the highest levels of IFN*α* ever measured in a viral illness, with concomitant high viral titer [116]. This elevation in IFN*α* levels is seen in mice infected with this virus, and seems crucial to the disease’s progression infection [262]. Similar high IFN*α* levels are observed in cell cultures infected with the new world arenavirus Machupo virus [263]. However, despite these elevated levels of IFN*α*, infections with multiple new world arenaviruses leads to the suppression of IFNβ expression [264, 265].

Lack of an early, balanced interferon response results in delayed immune activation and opportunity for viral replication during the early phases of infection. There is evidence that interferon response genes are upregulated early in infection with Lassa or Marburg viruses, suggesting that some subset of resistant cells may be crucial to responses [266]. In many of the HFVs discussed in this review (EBOV [5, 6], YFV [45], DENV [64]), high viremia is developed in early infections, with higher viremia in fatal cases and lower viremia in the later phases of infection marked by presence of viral reactive IgG.

### 4.2. Myeloid dysfunction

Myeloid cells, especially monocytes, macrophages, and dendritic cells, are commonly believed to be early sites of replication and systemic spread in hemorrhagic fevers. Monocytes circulate through the lung and other barrier tissues exposed in early infection and are among the first infected cells in EBOV and other conditions [20, 267]. While monocyte apoptosis is only seen in EBOV infections [35], infected myeloid cells are important sources of cytokines including TNF*α* and other factors such as NO that contribute to vascular integrity loss. *In vitro* modeling has shown that the secreted factors from infected monocytes or monocyte derived dendritic cells can drive loss of endothelial cohesion in EBOV [18, 267, 268], DENV, SNV [71], and Crimean-Congo HFV CCHV [269]. The exception to this pattern is LFV: monocytes infected with LFV fail to secrete proinflammatory cytokines even when stimulated with LPS [238].

Monocytes/macrophages also play a key role in HFV coagulopathy by producing procoagulants and fibrinolytics. Tissue factor (TF) is elevated in EBOV [42, 43], and in fatal SNV infection, lung macrophages secrete TF, uPA and PAI [145] with infected monocytes secreting abundant TF. Reversing TF activation in primate models of EBOV is therapeutic, which indicates the importance of this pathway in this disease.

Dendritic cell infection can compromise development of adaptive immunity, but infected dendritic cells are also sources of cytokines promoting endothelial activation and permeability [268–270]. Development of the high viremia seen in most of these infections can be related to delayed development of adaptive responses. In some cases, infection of dendritic cells can suppress activation of T cells [32–34, 101, 271, 272], though this is not universal [45, 71, 273].

### 4.3. Adaptive immunity

Adaptive immune responses comprised of neutralizing antibodies and reactive T cells play mixed roles in HFV infections. Development of adaptive immunity is crucial to viral control [180, 273–276]. When viral reactive IgM or IgG antibodies and T cells develop, viral titer tends to drop, and once the disease resolves, reinfection is unlikely. However, in contrast to this pattern, a previous infection with DENV increases the lethality of a subsequent infection with a different viral strain [277]. For the majority of pathogens discussed in this work, development of adaptive immunity is delayed (by effects on dendritic cells in CCHV), or suppressed (such as by the high numbers of regulatory t cells in CCHV [278], or immune checkpoint expression in EBOV [279]), permitting high viremia. Once high viremia is developed, an overwhelming response is necessary to clear the infection, potentially causing tissue damage in the process. Cytokine storm resulting in vascular leakage is a common feature.

While T cell responses are essential to resolution of most of the pathogens discussed, overactive T cells may promote vascular permeability and vasculogenic shock [280]. Importantly, leukocyte loss is observed in all the infections discussed in this work, along with altered ratios of leukocytes, such as the ratio of CD8 to CD4 cells, which tends to increase, and regulatory T cell (Treg) number which can decrease or increase [280]. While cytokine expression and T cell activity are crucial to survival, hypertrophic T cell responses can contribute to vascular leak and severity of illness. Notably, viral reactive T cell number correlates with severity of hantavirus infection [281], as does number of cytokine expressing cells found in the lungs. CD8+ T cell responses can damage the endothelia in both old world and new world hantaviruses [131, 282, 283]. Cytokines secreted by T cells such as TNF*α*, IL2, IL6 and IFN*γ* are found in hantavirus patients, and are known to contribute to capillary leak [281]. Likewise, some reports have linked severe LFV with nonspecific T cell activation [284] and a concomitant increase in disease severity at onset of T cell cytokine secretion is seen in DENV and YFV [285, 286]. Maintaining plasma volume during these phases of disease is challenging, contributing to lethality.

## 5. Microphysiological Systems to model vasculitis

Despite our growing understanding of HFV induced vasculitis, there remain key gaps in our understanding of these infections, and crucially, a lack of targeted therapies to improve survival. The complexity of HFV, the organ systems they affect, and the ranges of symptoms they invoke make these infections difficult to model. Current models include animal models and static 2D cell cultures, often fail to recapitulate human biology and the underlying complexity of molecular mechanisms of HFV infection [287]. Moreover, studies involving animal model are costly, time consuming, and complicated by species-specific susceptibility differences that hinder their translation to human biology [288]. While vaccines and therapeutic antibody treatments exist for some HFVs (e.g. YFV), in most of these infections, treatment is limited to supportive care. Importantly, there is a glaring absence of treatment strategies targeting the intrinsic vascular dysfunction associated with these infections.

### 5.1. Modeling HFV in MPSs

MPSs seek to model the function of human organ physiology [289], and offer great opportunity to study the molecular mechanisms behind vascular dysfunction in HFV-infected patients. While generally lower throughput than 2D systems and unable to recapitulate the physiology of an entire organism, MPS provide several distinct advantages, including tunability and flexibility, medium-throughput, and cost-effectiveness relative to animal models [290]. MPS can be tailored to incorporate different cell types, can have different geometries to build more complex systems such as multi-layer devices containing endothelial and epithelial layers, and can incorporate different mechanical stimuli via interstitial and vascular flow.

**Figure 3.**
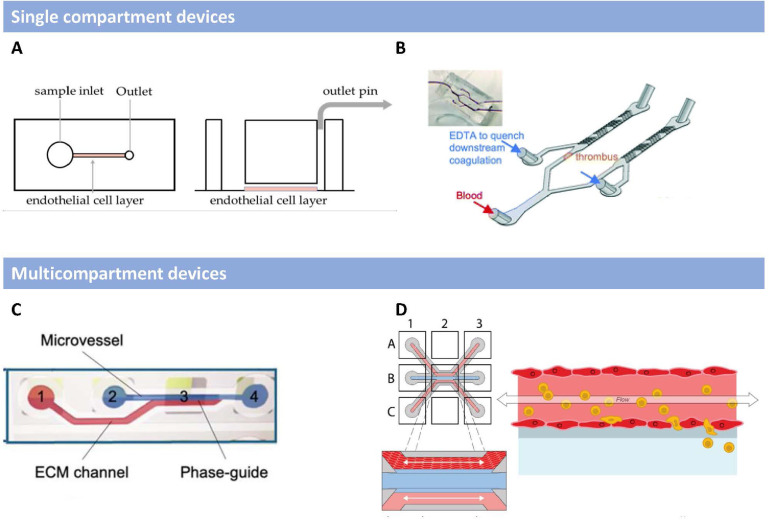
Examples of microphysiological systems by categories. The MPS have been grouped in two categories based on their complexity, and number of compartments. (A), (B) Devices shown in A [294] and B [298] are representative MPS grouped in the single compartment MPS category. Reproduced from [294]. CC BY 4.0. Reproduced from [298]. CC BY 3.0. These devices mainly consist of a single tissue channel perfused by blood or media to study vasculitis. (C), (D) Devices shown in C [291] and D [320] highlight examples of the types of devices grouped in the multicompartment category, these devices feature two or more channels and cells in these MPS are usually subject to interstitial flow. Reproduced from [291]. CC BY 4.0. © 2020 The Authors. Biotechnology and Bioengineering Published by Wiley Periodicals LLC. Reproduced from [320]. CC BY 4.0.

To date, only a few studies have used MPS to study HFV [291, 292], which represents a major opportunity to develop and utilize MPS to better understand HFV as well as develop effective countermeasures. There are many studies that have utilized MPS to model one or more components of the ‘vasculitis triad’: immune dysregulation, endothelial dysfunction, and clotting (table 2) in a different context than HFV. These MPS have been divided into two different groups based on their complexity: (1) single compartment and (2) multicompartment devices. The first group is composed of devices that mainly consist of a single microfluidic channel lined with endothelial cell monolayers, generally human umbilical vein endothelial cells (HUVECs), in type I collagen or fibrin (figures 3(A) and (B)). The endothelial cells are exposed to a wide range of shear stresses (1–40 dyne cm^−2^). Despite the relative simplicity of these devices, some studies have included all features of the vasculitis triad [293–295]. In these devices the endothelial cells are pre-conditioned with TNF*α* (to model vascular dysfunction), followed by perfusion with whole blood to evaluate clotting and immune cell activation and recruitment. Two additional MPS in this category include studies by Lu *et al* [296] and Rajeeva Pandian *et al* [297] which evaluate the recruitment of peripheral blood mononuclear cells (PBMCs) and clotting, respectively, upon exposure of HUVECs to SARS-CoV-2. Although simple in design, these devices have provided important insight into vascular dysfunction, immune cell recruitment, and clotting of microvessels in response to a virus providing a platform for the study of HFV. However, a limitation of these MPS are a lack of interstitial flow (MPS in this category are exposed to 1D flow), and surrounding stroma (cells and matrix).

**Table 2.** Microphysiological systems to investigate the vascular triad of HFV.

Device classification								
Group 1- single component devices	Immune cells	Vascular inflammation	Clotting	Vascular endothelium	Epithelium	Stroma	Endothelial shear stress (dyne cm^−2^)*	Reference
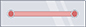	PBMC	HCoV-NL63		HUVECs		Gelatin	1.3–1.4	[296]
		SARS-CoV-2 cytokines	Platelets, fibrin (WB)	HUVECs		Collagen-fibronectin	2	[297]
	Leukocyte (CD45)	TNF-*α*	Platelets (WB)	HUVECs		Fibrinogen	Wall shear stress: 2–20; stenosis region: >100	[293]
	Neutrophils	TNF-*α*	Platelets fibrin (WB)	Fixed HUVECs		Fibronectin	1–6	[294]
	Leukocytes		Platelets, fibrin (WB)				40	[298]
	THP-1 cells	TNF-*α*		HUVECs		Fibronectin, HASMCs	2.4	[299]
			Platelets, fibrin (WB)	HUVECs		Collagen and tissue factor (TF)		[300]
		TNF-*α*	Platelets (WB)	HUVECs		Type I collagen	40	[301]
		TNF-*α*	Platelets, fibrin (WB)	HUVECs		Type I collagen, fibronectin	2	[302]
	Leukocytes	TNF-*α*	Platelets, fibrin (WB)	BOECs		Type I collagen	25	[295]
		MASP-1	Fibrin (WB)	HUVECs		Fibronectin	1–4	[303]
			Platelets, fibrin (WB)	HUVECs		Type I collagen	40	[304]
			Fibrin (WB)			Fibronectin		[305]
Group 2-multicompartment devices	Immune cells	Vascular inflammation	Clotting	Vascular endothelium	Epithelium	Stroma	Endothelial shear stress (dyne cm^−2^)*	Reference
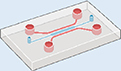		Ebola VLP		HUVECs		Type I collagen		[292] ^#^
		Lassa VLP		HUVECs		Type I collagen		[291] ^#^
	Neutrophils	Influenza H1N1, H3N2, H5N1 strains, SARS-CoV-2 VLP		HLMVECs	HBEpCs	Collagen type IV.		[306]
	Macrophages U937	Poly(I:C). SARS-CoV-2_del19AA-GFP pseudovirus		HUVECs	ihAEpiC	Type I collagen		[307]
	Macrophages	SARS-CoV-2		HUVECs	Calu-3 cells			[308]
	Macrophages	SARS-CoV-2		HLMVECs	ATs	Type I collagen, fibronectin		[309]
	PBMCs	SARS-CoV-2		HULEC-5a	Vero E6, ihAEpiC	Type I collagen		[310]
	PBMC	SARS-CoV-2		HUVECs	Caco-2, HT-29	ECM membrane		[311]
		Plasma from patients infected with SARS-CoV-2		HUVECs		Type I collagen	1–5	[312]
		SARS-CoV-2 subunit S1		hBMVECs		Type I collagen, hyaluronan, and Matrigel	0.7	[312]
		SARS-CoV-2 pseudo-virus and diabetic human plasma		HUVECs		Fibronectin	0–0.1	[313]
	Neutrophils	TNF-*α* and fMLP		HUVECs		Fibronectin	<20	[314]
	Leukocytes (WB)	TNF-*α*	Platelets (WB)	HLMVECs		Fibronectin and dextran for cell adhesion	1–4	[315]
	Leukocytes	TNF-*α*	Platelets, fibrin	HUVECs		Fibronectin	5	[316]
	Neutrophils (CD66b+) and monocytes (CD14+)	TNF-*α*	Platelets	HUVECs			1–4	[317]
	Neutrophils	TNF-*α*	Platelets, fibrin	HUVECs		Fibronectin	5	[318]
	THP-1	TNF-*α*, IL-1B		HAoEC		Type I collagen, Matrigel, HAoSMC		[318]
	PBMC	TNF-*α*, IFN-*γ*		HUVECs		Type I collagen		[319]
	CD3+ T cells	TNF-*α*		HMEC-1	A375	Type-I collagen		[320]
	Neutrophils	IL-13		HLMVECs	HAECs	Type I collagen	1	[321]
	THP-1	LPS		HUVECs		Type I collagen		[322]
		TNF-*α*	Platelets	HUVECs		Extracellular matrix-coated membrane	5	[323]
		TNF-*α*, LPS inflammation	Platelets	HUVECs		ECM-coated porous membrane	2.5–7	[324]
		TNF-*α*	Platelets, fibrin	HUVECs fixed		Type I collagen	7	[325]
	Leukocytes		Platelets, fibrin	HUVECs	HBVPCs	Type I collagen, HUASMCs	0–10	[326]
			Platelets, fibrin (WB)	HUVECs		Gel MA, fibroblasts		[327]
		Vascular injury by puncture to microvessel	Platelets, fibrin (WB)	HUVECs		3D collagen hydrogel + TF	4	[328]

Footnote:.

BOECs = Blood outgrowth endothelial cells.

HAECs = Human airway epithelial cells.

HAoEC = Human aortic endothelial cells.

HAoSMC = Primary human aortic smooth muscle cells.

HASMCs = Human aortic smooth cell muscle cells.

HBEpCs = Primary human bronchial epithelial cells.

hBMVECs = Human brain microvascular endothelial cells.

HBVPCs = Human brain vascular pericytes.

HLMVECs = Human lung microvascular endothelial cells.

HUASMCs = Human umbilical arterial smooth muscle cells.

HUVECs = Human umbilical vein endothelial cells.

ihAEpiCs = Immortalized human alveolar epithelial cells.

PBMCs = Peripheral blood mononuclear cells.

WB = Whole blood.

VLPs = Virus-like particles.

TF = Tissue factor.

*Values have been converted to shear stresses, assuming blood viscosity = 0.004 Pa*s for those devices perfusing blood and 0.0089 Pa*s for those using media.

MPS figures obtained from BioRender.

# = MPS studying HFV.

MPS in the second group are more numerous and these systems mimic more complex phenomena by including adjacent channels or layers (i.e. multicompartment) that allow for interstitial flow, the transport of molecules, and cells within different compartments and in different directions (figures 3(C) and (D)). These devices can contain a tissue-specific epithelium (e.g. lung) to mimic the interactions of viruses between the vascular and stromal compartments. The adjacent layers and channels in these MPS allow for molecules to migrate within different spaces and interact with neighboring model tissues. The added complexity of these MPS bear enhanced physiological relevance to human (patho)physiology and can thus potentially be used to study the interactions of hemorrhagic viral particles with both the endothelium and neighboring stroma. Examples of these devices include those by Si *et al* [306] and Cao *et al* [307] which utilize multicompartment devices containing one channel seeded using endothelial cells and adjacent channel coated with bronchial basal cells and alveolar epithelial cells respectively to study how SARS-CoV-2 circulating through the epithelium can trigger vascular dysfunction and immune cell recruitment.

MPS are promising to investigate viral hemorrhagic infection at the molecular level [329] and for the rapid testing of drug candidates to treat these diseases. Despite the lack of MPS used to study HFV [89, 130], other systems presented in table 2 could easily be adapted for study the molecular mechanisms underlying HFV-induced vascular dysfunction. These MPS include those by Tsai *et al* [315], Greineder *et al* [316] and Venugopal Menon *et al* [317] which contain channels coated with endothelial cells and are inflamed with TNF*α* to evaluate immune cell recruitment, clotting and vascular dysfunction.

Generally, MPS systems offer many advantages over 2D *in vitro* and animals models due to their dynamic (allowing temporal measurements), tunability (enabling interchangeability of multiple cell types), and potential cost-effectiveness. As such, they offer the possibility to discover new HFV interventions. Importantly, most assays performed on patients can be used in MPS, though with some adaptation to the limited size and limited physiological complexity of MPS systems (table 3). Additionally, many of these platforms can be purchased commercially (e.g. MIMETAS, Emulate, InSphero).

**Table 3.** Assays.

	Clinical/ *in vivo* assays	*Ex vivo* assays	MPS/*in vitro* assay
Endothelial dysfunction			
Glycocalyx	• Soluble HA ELISA or multiplex immunoassays	• Electron microscopy on vascular apical surface • Lectin staining for glycocalyx components	• Electron microscopy on vascular apical surface • Lectin staining for glycocalyx components
Barrier function & junctions	• Hematocrit for hemoconcentration • Loss of serum albumin • Formation of petechiae	• Immunostaining for tight junction components (Zo1, Occludin, Claudins) • Immunostaining for adherens components (VE-Cadherin)	• Permeability of tracer molecules. • Immunostaining for tight junction components (Zo1, Occludin, Claudins) • Immunostaining for adherens components (VE-Cadherin)
Basement membrane organization		• Immunostaining for BM components (Laminins, Collagen IV, Collagen VI)	• Immunostaining for BM components (Laminins, Collagen IV, Collagen VI) • Non-canonical amino acid tagging for nascent protein secretion
Endothelial cell health and cell activation	• ELISA/multiplex ELISA for sICAM, sVCAM, sCD62P/L, sCD31	• Expression of endothelial specific markers, CD31, vCAM, and transcription factors KLF2/4 • Expression of endothelial activation markers ICAM, VCAM1, CD62P/L • Endothelial transcriptomics	• Cell and cytoskeletal alignment with direction of flow • NO secretion • KLF2/4 expression • Endothelial transcriptomics • Myeloid attachment assays • Cell growth and contact inhibition assays
Coagulopathy			
Coagulation	• Assays for total fibrinogen, clotting system components, D-dimer, PTT,		• Coagulation on chip assays/deposition of labeled fibrinogen
Platelets	• Platelet counts • Platelet function assays • Platelet FACS		• Platelet counts • Platelet function assays • Platelet FACS
Immune dysregulation			
Interferon system	• ELISA/multiplex ELISA for interferons		• Interferon secretion assays • Interferon time course dynamics • Cell supernatant cell activation assays
Myeloid	• PBMC flow cytometry • PBMC transcriptomics • Cytokine/chemokine multiplex ELISA panels on serum		• Myeloid cell flow cytometry • Myeloid cell transcriptomics • Cytokine/chemokine multiplex ELISA panels on effluent • Myeloid attachment to endothelia assays
Adaptive immunity	• IgM/IgG titers for antibodies • Antibody neutralization assays on serum • Tetramer assays for reactive T cells		• Infection and antibody neutralization assays • *In vitro* cytotoxicity assays

Two rapidly advancing technologies have been underappreciated by MPS systems attempting to mimic the vascular triad. The first is 3D printing, which has seen a steady improvement in resolution over the past decade. Some light-based bioprinting systems can now achieve features smaller than 100 *μ*m [330], rivaling soft lithography. Furthermore, the cost of 3D printers has been steadily declining, making this technology widely accessible to a range of researchers. The second technology is gene editing using CRISPR-Cas or related systems. These technologies enable rapid and efficient modification of the genome of cell lines or primary cells [331], facilitating the attainment of altered or desired phenotypes that better reflect the inflammatory niche of the vascular triad. In summary, by leveraging microfluidic devices to establish microenvironments that mimic human (patho)physiology, researchers can study the behavior of cells and tissues in more physiologically relevant environments.

### 5.2. Challenges to modeling HFV in MPSs

Despite the potential of MPS to mimic features of human (patho)physiology, numerous challenges exist; some specific to the vascular triad and others more specific to MPS, in general. For example, one of the greatest challenges to MPS, in general, is appropriate cell sourcing for realistic and repeatable simulation of human physiological phenomena. While primary cells are generally the first choice, harvesting primary human cells has challenges related to access, cost, purity, limited lifespan (i.e. limited passaging), and inter-subject variability. As such, most studies include samples from only a small number of human subjects making broad conclusions about the human response difficult.

Another significant challenge involves co-culturing of multiple cell types within a single microphysiological niche. Typically, most cell types, especially primary cells, require an optimized cell culture medium. Therefore, combining two or more cell types necessitates the blending of multiple media, each of which has been optimized for only a single cell type. Developing optimized cell culture medium for multiple cell types is time consuming and costly, may result in altered cell phenotypes that do not reflect the *in vivo* environment, and generally includes more than one endpoint for which the optimal condition is created.

A final major hurdle, particularly pertinent to HFV and the vascular triad, is replicating the intricate dynamics of the human immune response. Specifically, the adaptive immune response entails antigen presentation, involvement of multiple tissues (e.g., lymph nodes), various cell types (e.g. T cells, B cells), and appropriate time for development following antigen exposure. While these hurdles are all significant, they are not insurmountable. The general strategy to overcome these obstacles is to build models gradually, carefully delineating features of the MPS that are included as well as those that are not. The microfluidic models of the vasculitis triad thus far (table 2) have the potential to study mechanisms of HFV and thus to identify new treatments for these diseases.

## 6. MPS beyond HFV: modeling vascular dysfunction in SARS-CoV-2 infection

MPS that allow for the study of elements of the triad have broader impact beyond studying HFV. Many infectious pathogens can trigger a vascular response (e.g. vasculitis) either indirectly (e.g. infectious agents that invoke an immune response) or directly by injuring or activating the endothelium, both mechanisms leading to potentially devastating systemic consequences [1]. Invasion or infection of endothelial cells by viral or bacterial pathogens is common (e.g. *Escherichia coli, Salmonella typhi*, Herpes Zoster, Polioviruses, Measles Virus etc) and can result in severe complications like thrombosis, causing heart attacks or stroke, or vascular leakage which can cause hypovolemic shock [332]. The innate immune response triggered by these infection acts as the initial defense against pathogens, however, this response can also damage the endothelium [333], exacerbating infection-induced morbidity. This was most notably seen recently in severe SARS-CoV-2 infection, where the occurrence of ‘cytokine storms’ or excessive release of inflammatory mediators was associated with increased mortality rates [334].

The necessity for human-relevant models to study viral tropism, especially those encompassing a vascular system, was exemplified particularly during the SARS-CoV-2 pandemic. Initially perceived as primarily targeting the respiratory system akin to prior coronaviruses, COVID-19 patients early on presented extensive coagulation issues and signs of endothelial dysfunction (endothelilopathy and endotheliitis) [335]. SARS-CoV-2 is known for its binding affinity to cells expressing hACE2 receptor, prevalent across diverse human organs including lung, heart, kidney and vasculature [336, 337]. Initial clinical and post-mortem data implicated vascular involvement and direct infection of endothelium was speculated [338, 339]. However, animal studies were insufficient in providing further insight into understanding virus-vasculature interactions. Though transgenic mice expressing hACE2 in lung epithelium were susceptible to SARS-CoV-2 and used as infectable COVID19 animal models, these animals lack the ACE2 receptor in their vasculature. Consequently, effects of SARS-CoV-2 infection in organs like heart, spleen, or kidney [336, 337] were not observed. Similarly, animal models like macaques and hamsters displayed susceptibility to infection but failed to exhibit the coagulation abnormalities or cytokine storm observed in human patients due to species differences in the ACE2 receptor. Conventional static 2D cell cultures were not able to replicate direct infection [340, 341] or demonstrated limited replication [342] in endothelial cells. Furthermore, these static cultures of endothelial cells did not show disruption of barrier function nor were inflammatory response observed [343].

Recent data emerging from MPS that incorporated all or some of the components of the triad have helped elucidate the pathogenesis of COVID-19 vasculopathy. Unlike HVF where coagulopathy dysregulation induces hemorrhaging, thrombosis is a result of perturbation to the endothelium and immune dysregulation caused by SARS-CoV-2 infection. In a recent MPS of a human alveolus, Zhang *et al* [310] were able to replicate the inflammatory response arising from direct infection of lung epithelium cells and demonstrated vascular dysfunction as well as recruitment of immune cells. Similarly, in another MPS focused on intestinal epithelium-vascular endothelium, again endothelial cells were not found to be susceptible to viral infection but did exhibit changes in barrier function after exposure to SARS-CoV-2 and transcriptional analysis revealed activated immune response in endothelial after viral infection [311]. These more nuanced interactions arising from these complex MPS highlight the promise and need for these models for not only better understanding of pathobiology of vasculotropic pathogens but as potential testbeds for developing countermeasures. The MPS can serve as a useful intermediary between *in vivo* animal studies and clinical data.

## 7. Future outlook

According to the Disease Outbreak News, a public online reporting system managed by the World Health Organization, HFV outbreaks continue sporadically, posing a public health threat for individuals across the world. Although treatments for some of these diseases exist, they primarily consist of supportive care to mitigate complications in patients [332]. Current treatment strategies largely derive from insights gleaned through animal models and 2D cultures. These models, though simplistic, have significantly expedited the development of diagnostics and vaccinations. However, there is a need for more comprehensive solutions to treat and prevent the multisystem effects of these diseases. Current HFV *in vitro* models generally lack the complexity necessary to study the molecular interactions between the virus and host to study the vascular triad characteristic of HFV. MPS, on the other hand, provide a pathway to explore the myriad of mechanisms that underlie human infection to viruses that lead to HFV. A deepened understanding of these mechanisms could drive the development of new prophylactic measures and treatments.

Despite the significant medical complications of HFV, only a limited number of studies [293, 294] utilize MPS to examine vascular leakage (hemorrhage) during HFV infection, underscoring the need for additional platforms. These studies employ a single-compartment MPS lined with endothelial cells which were then exposed to LFV and EBOV virus, respectively. Post-exposure vascular permeability was measured to assess the extent of leakage upon infection. In both studies, the introduction of LFV and EBOV virions led to an elevation in vascular permeability and increased formation of actin filament stress fibers. This leakage correlated with the overactivation of the Rho/ROCK pathway, known for its role in cytoskeleton remodeling. In the EBOV HFV model, the loss of vascular integrity was rescued using FX06 and melatonin, indicating their therapeutic potential for treating EBOV. These studies, while successful in demonstrating the efficacy of FX06 and melatonin in rescuing vascular integrity, are merely scratching the surface in developing a comprehensive mechanistic understanding of virus-induced vascular leakage. For instance, MPS could delve into molecular-level viral entry mechanisms like the CD55 entry mechanism, common among various bloodborne viruses, including PUUV [220], or serve as a platform for testing drugs to restore vascular balance.

Limited access to BSL-4 facilities has certainly hindered the use of HFV in MPS. As previously mentioned, (table 2), there are various studies that incorporate elements of the triad characteristic of HFV infections. Utilizing these devices could be effective at simulating HFV-induced symptoms and studying disease progression, without involving virus handling in BSL-4 facilities.

HFV often affect multiple organs including the heart, kidneys, and liver, causing severe bleeding and even death [344]. Progress in the field of multi-organ-on-a-chip systems holds great promise to better understand the systemic effects of these viruses on different organs. These multi-organ MPS promise to integrate different organs such as brain, pancreas, bone, skin, liver, lung, heart, gut, and endometrium, as recently demonstrated by Wang *et al* [345]. In this study, a multi-organ MPS was constructed to evaluate the response of these organs to Tolcapone, a drug to treat Parkinson’s disease. A multi-organ MPS could replicate the complex interplay of HFV including multi-organ failure as observed with EBOV. Moreover, these systems could potentially evaluate treatment effectiveness in controlling or preventing infections, expediting drug development and aid in understanding diseases causing vascular dysregulation including SARS-CoV-2.

MPS hold great promise for accelerating the development of drugs to effectively treat complications that may stem from HFV infections as well as other diseases that lead to vascular dysregulation, including SARS-CoV-2. These systems can replicate specific features of 3D human physiology, enabling more precise and reliable testing for prospective drugs or therapeutic interventions for HFV infections. Finally, MPS can be used to examine disease progression and be customized to simulate patient-specific physiology, thereby facilitating the design of personalized treatments.
